# Deposition and Properties of Nanostructured Multilayer Cr/(Cr/a-C)ml Coating on Stainless Steels

**DOI:** 10.3390/ma18245654

**Published:** 2025-12-16

**Authors:** Valentin Mishev, Yavor Sofronov, Milko Yordanov, Antonio Nikolov, Krum Petrov, Rayna Dimitrova, Milko Angelov, Boyan Dochev, Krassimir Marchev, Georgi Todorov

**Affiliations:** 1 Department of Material Science and Technology of Materials, Faculty of Industrial Technology, Technical University of Sofia, 1756 Sofia, Bulgaria; anikolov@tu-sofia.bg (A.N.); kpetrov@tu-sofia.bg (K.P.); r_dimitrova@tu-sofia.bg (R.D.); 2 Department of Theory of Mechanisms and Machines, Faculty of Industrial Technology, Technical University of Sofia, 1756 Sofia, Bulgaria; 3 Department of Mechanical Engineering, Manufacturing Engineering and Thermal Engineering, Faculty of Engineering and Pedagogy—Branch Sliven, Technical University of Sofia, 8800 Sliven, Bulgaria; m_yordanov@tu-sofia.bg; 4 Faculty of Industrial Technology, Technical University of Sofia, 1756 Sofia, Bulgaria; milko.angelov@tu-sofia.bg (M.A.); k.marchev@northeastern.edu (K.M.); 5 Department of Mechanics, Faculty of Mechanical Engineering, Technical University of Sofia—Branch Plovdiv, 4000 Plovdiv, Bulgaria; dochev@tu-plovdiv.bg; 6 College of Professional Studies, Northeastern University, Boston, MA 02115, USA; 7 Center of Excellence “Mechatronics and Clean Technology”—Campus Studentski Grad, Technical University of Sofia, 1756 Sofia, Bulgaria; gdt@tu-sofia.bg; 8 Department of Manufacturing Technology and Systems, Faculty of Industrial Technology, Technical University of Sofia, 1756 Sofia, Bulgaria

**Keywords:** nanostructured multilayer coating, Cr/(Cr/a-C)ml coating, mechanical properties, wear resistant stainless steel, nanostructured multilayer coatings, coating mechanical properties, stainless steel, magnetron sputtering deposition, functional coating

## Abstract

This work presents the results of deposition by magnetron sputtering nanostructured multilayer Cr/(Cr/a-C)ml coatings on AISI 316L and AISI 321 steel substrates. Chemical compositions were confirmed through EDX analysis with scanning electron microscopy. The coating thickness was measured with Calotester (KaloMAX II) and the total thickness of the coatings obtained ranged from 1.684 ± 0.193 μm for AISI 316L to 1.749 ± 0.123 μm for AISI 321. A Daimler-Benz Rockwell indentation test for adhesion quality and a nanoindentation test with a Berkovich indenter were carried out. According to the Raman spectroscopy analysis and in agreement with mechanical tests, it is supposed that it is the formation of a diamond-like carbon phase which enhances the mechanical properties. The hardness values obtained for the nanostructured multilayer Cr/(Cr/a-C)ml coatings were improved compared to those of the base stainless steels.

## 1. Introduction

Stainless steels have wide applications in almost all areas of human activity, which is due to a combination of their three most important properties—strength, plasticity (formability) and corrosion resistance. Out of these three, the most important property is corrosion resistance, which is attained by alloying with Cr above 11%. In order to improve some of the mechanical or anti-corrosion properties of these steels, they may be additionally alloyed with elements such as Ni, Ti, Mo, Si, Nb, Al, Cu and N. They are available in the form of rods, sheets, strips, plates, foil, wire, semi-finished products, pipes and tubes [1,2,3,4,5].

Due to their good service characteristics and wide applicability, the worldwide consumption of stainless steel increases constantly. For the period 2024–2030, the market consumption of stainless steel is expected to increase from USD 120.2 billion in 2024 to USD 157.4 billion in 2030, a growth at a CAGR of 4.6% from 2024 to 2030 [6].

Stainless steel owes its corrosion resistance to a thin passivated layer of chrome oxide or hydroxide, which forms from interaction between the steel and its environment—liquid or gaseous. This film protects items made of stainless steel from aggressive compounds in the environment. It is very thin (a few atomic layers) and with friction or just from physical contact with another solid body, it often results in surface degradation of the metal part, known as pitting or flaking [7]. In addition to this, modern construction components and tools made of conventional or stainless steel face ever-increasing or completely new service requirements [5]. For this reason, many researchers worldwide are developing new means to improve the mechanical properties and corrosion resistance of stainless steel objects, by grain size refinement, the application of coatings, the creation of diffusion layers, or the addition or change of alloying elements in the steel, among others [5,8,9,10].

Austenite stainless steel AISI 316L/EN 1.4404 and AISI 321/EN 1.4541 [11,12] are important samples, because as well as strength, plasticity, and corrosion resistance at room temperature, they retain their corrosion resistance and strength at much higher temperatures—steel 316L up to 857 °C and steel 321 up to 900 °C, but they do not have sufficiently high surface hardness or wear resistance [13,14,15]. For this reason, these types of steel are the objects of investigation in the current paper.

One of the widely used means for protecting the surface of stainless steel, which derives from mechanical interaction, which also leads to decreased corrosion resistance, is to apply thin protective coatings. In the last decade, it has become common to apply different types of hard carbon coatings—such as non-doped diamond-like carbon (DLC)—as well as hard carbon coatings alloyed with different elements such as Ti, Cr, W, Mo, Cu, Si, Ce and others (doped DLC coating). DLC coatings possess high hardness, wear resistance, a low coefficient of friction, high corrosion resistance, heat resistance and biological compatibility [16,17,18,19,20,21].

The authors [19,22,23] have established that Cr-alloyed hard carbon coatings exhibit high hardness, wear resistance, and corrosion resistance, which are influenced by the temperature of deposition and the coating’s composition—alongside the quantity of Cr and the presence of amorphous a-C phase in it. The authors [23] determine that, in particular, nanostructured hard Cr-C films are suitable for coating against oxidation of stainless steel electrodes. This way improves the properties of the working surfaces of parts made of stainless steel. This provides a good reason, the focus of the current paper, for the application of nanostructured multilayer Cr-doped hard carbon coatings over samples of steel AISI 316L and AISI 321.

The production and deposition of thin wear-resistant and corrosion-resistant coatings using physical vapor deposition (PVD) methods have found wide applications in all areas of industry. Research continues obtaining new combinations and structures of coatings, as well as improvements in the technologies for their deposition by the magnetron sputtering method on metals and non-metals. Among the wide variety of thin coatings deposited by PVD methods, extensive research is being conducted on carbon-containing coatings, including chromium–carbon coatings [24,25,26,27,28].

The aim of the present work is to investigate the possibility of deposition by non-reactive magnetron sputtering of nanostructured multilayer chromium–carbon hard coatings on austenitic stainless steels, to represent their base structural and mechanical parameters and possibilities for application of these coatings.

## 2. Materials and Methods

### 2.1. Materials

Two grades of austenitic stainless steel—AISI 316L (X2CrNiMo17-12-2/1.4404 EN 10088-1:2024 [29]) and AISI 321 (X6CrNiTi18-10/1.4541 EN 10088-1:2024) with dimensions of 20 × 20 × 5 mm—were studied. The chemical compositions of the steels are represented in Table 1.

Both steel grades are non-magnetic and are designed for improved performance under corrosion and temperature conditions than standard stainless steels (AISI 304).

Steel grade 316L is the low-carbon version of 316; it is resistant to sensitization (carbide precipitation at grain boundaries) and is widely used in thick-walled welded components. Grade 316L steel retains its strength and resistance to corrosion in acids and bases at high temperatures thanks to its chromium content (around 17%), increased nickel content (around 12%), and molybdenum content of 2% to 3%. Their austenitic structure gives these grades excellent impact toughness, even at cryogenic temperatures, improved creep resistance, and improved tensile strength at elevated temperatures compared to a range of other stainless steel grades. Steel grade 316L is considered resistant to potable water with up to around 1000 mg/L chlorides at ambient temperatures, reducing to about 500 mg/L at 60 °C [30,31].

Steel grade 321 is a basic austenitic steel (AISI 304) which is stabilized by titanium additives. This grade is used as it is not susceptible to intergranular corrosion after heating within the carbide precipitation range of 425–850 °C. Steel 321 is used for applications in a temperature range up to about 900 °C, combining high strength, resistance to scaling, and phase stability with resistance to subsequent aqueous corrosion. The alloy is subject to pitting and crevice corrosion in warm chloride environments and to stress corrosion cracking above 60 °C. It is considered resistant to potable water with up to about 200 mg/L chlorides at ambient temperatures, reducing to about 150 mg/L at 60 °C [30,31].

Both stainless steels are widely used in the petroleum, chemical, food, and pharmaceutical industry, in building, automotive, and power plant constructions as well as in marine applications, kitchen appliances, and in medicine for instruments and implants, etc., [30,32].

### 2.2. Preparation and Deposition of Coatings

The preparation of the substrate surfaces before magnetron sputtering is essential for the adhesion of the coatings. Before coating deposition, both steel substrates were prepared by successive grinding with silicon carbide abrasive papers from grade 200 to grade 1600. Polishing was carried out with a 5 µm diamond polishing agent (Struers GmbH, Willich, NRW, Germany). After polishing, the surfaces were cleaned with an ultrasonic bath with deionized water and degreased in an ultrasonic bath with Deconex^®^ cleaning reagent (4% dissolved in deionized water, Borer Chemie AG, Zuchwil, Switzerland) at 60 °C for 10 min, with subsequent washing in deionized water, drying by air blowing, and final drying for 1 h in a vacuum dryer at 100 °C. All this preparation of the substrates was carried out up to 1 h before they were placed in the chamber of the PVD system to begin the magnetron deposition process.

**Figure 1:**
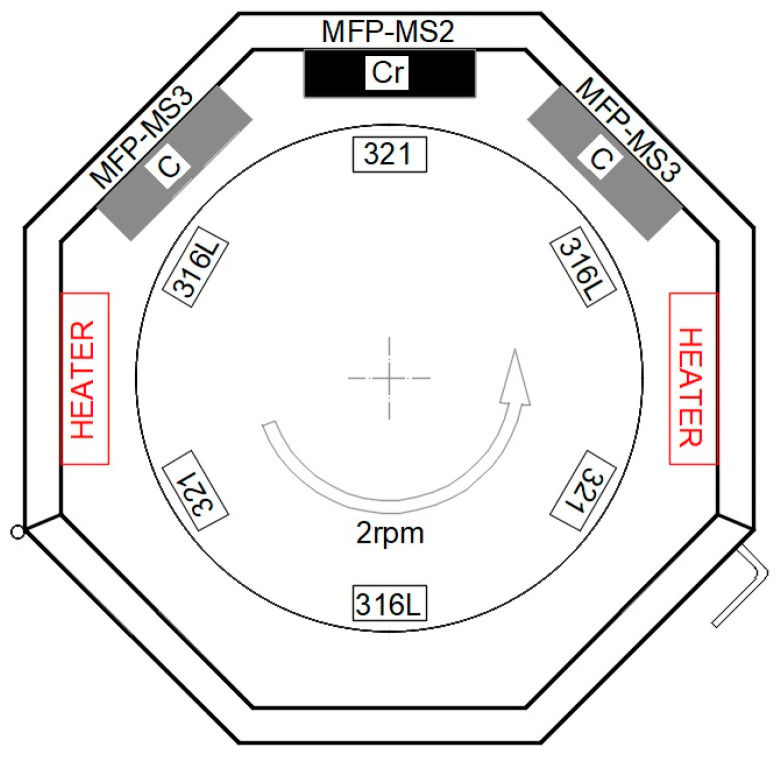
PVD system for the magnetron deposition of coatings.

A nanostructured multilayer Cr/(Cr/a-C)ml coating was deposited on to the substrates of 316L and 321 stainless steels. The coating process was carried out in a magnetron deposition system with three unbalanced rectangular magnetrons and a rotating table. The block diagram of the original patented design (BG67500B1) [33] of the PVD system is shown in Figure 1.

The PVD camera is an octagonal chamber with three unbalanced magnetrons equipped with rectangular targets and a single-axis rotating table for substrate mounting. This PVD system works with rectangular targets with dimensions of 360 × 102 × 9 mm. Due to the significantly lower speed of magnetron sputtering of carbon, compared to the speed of sputtering of chromium [34,35,36], the coatings were obtained by simultaneous sputtering of three targets, two of which were carbon with a purity of 99.99% and the third of which was chromium with a purity of 99.8%. During coating deposition, the substrates were placed at a distance of 60 mm from the targets on a rotating table with a vertical axis of rotation, which rotated at 2 rpm [37].

The process of magnetron sputter deposition of the Cr/(Cr/a-C)ml nanolaminate multilayers involved five stages: (1) turning on the rotation of the table with the substrates at 2 rpm and cleaning of substrates by glow discharge at 4–5 Pa and 250 °C in an Ar+H_2_ mixture at −900 V MF pulsed bias for 15 min; (2) cleaning of the substrates with chromium ions at 3 × 10^−1^ Pa, 1000 W on the chromium target and −900 V bias at 250 °C for 15 min; (3) deposition of the 150–250 nm thick chromium sublayer at 2.6 × 10^−1^ Pa and chromium target power of 1850 W for 8 min; (4) to reduce the internal stress in the multilayer coating, a gradient chromium–carbon intermediate layer was deposited first by applying 250 W to both graphite targets for 1 min and then by gradually increasing their power to 750 W and reducing the power of the chromium target to 700 W for 10 min; and (5) deposition of a Cr-C nanostructured multilayer for 180 min at 2.6 × 10^−1^ Pa with an Ar 160 °C substrate temperature, −90 V substrate bias voltage, 700 W chromium target power, and a power for the two carbon targets of 750 W each. Six samples were produced using these PVD system settings—three from each stainless steel grade.

### 2.3. Methods of Investigation of Coatings

The method for the application of thin coatings by magnetron sputtering possesses many advantages, the most important of which are as follows:

- Reactive and non-reactive application of coatings;
- Ability to make mono- or multilayered coatings;
- Wide temperature range of the substrate during coating application processes (from room temperature to 700 °C);
- Sputtering from metal or non-metal targets;
- Application of coatings over metal or non-metal substrates, such as glass, polymers, and textile canvas of natural or artificial origin;
- Ability to apply layers with different compositions and thicknesses;
- The method does not require oxidizing or corrosive environments;
- It is ecological;
- It is suitable for application on an industrial scale, allowing for introduction of a high level of automation in the process in continuous production lines.

All this makes it suitable for application of Cr-doped nanostructured multilayered coatings of hard diamond-like carbon over different substrates.

The methods for the investigation of the coatings in this paper involve the following assessments, so we can determine their principal characteristics as being solid, Cr-doped DLC, with suitable adhesion to the substrates. These assessments comprise

- Measurement of the coating’s thickness by the Calotest method;
- Surface morphology assessment by SEM;
- Adhesion test by Rockwell C;
- Assessment of the chemical composition by SEM-EDS;
- Measurement of hardness and Young’s Modulus by nanoindentation;
- Assessing the structural characteristics of the of intensity ratio between *I_D_/I_G_* by Raman spectroscopy.

To evaluate the magnetron sputtering process used for obtaining the nanostructured multilayer coating of Cr/(Cr/a-C)ml on AISI 316L and AISI 321 stainless steels, a series of analyses and tests were performed to determine their structural characteristics and mechanical properties.

The coating thicknesses on both steel grades and the layers therein were determined according to EN ISO 26423:2016 [38], using the “KaloMAX II” calotester from BAQ GmbH (Berlin, Germany), with the test parameters represented in Table 2. The composition of abrasive slurry is 1 µm grain size diamond paste suspended in ethanol with a concentration of 1:4 (BAQ, Germany).

The adhesion of the coatings was determined according to EN ISO 26443:2024 [39]. Since the measured Rockwell hardness in both steel substrates (316L and 321) is below 20 HRC, the test was performed by Rockwell A scale (120° diamond cone, 200 nm tip radius, 60 kgf/588.4 N) on a VEB WPM Leipzig (Leipzig, Saxony, Germany) hardness tester. The crater for the thickness and indentation for the adhesion of the coatings was measured using a Best Scope BS-6022TRF (Shenzhen, China) microscope and Capture 2.1 software. Reports were prepared in accordance with the standards for testing thickness (EN ISO 26423:2016) and adhesion (EN ISO 26443:2024) of the obtained coatings. Surface nanostructure and chemical composition of the coatings were analyzed via scanning electron microscopy EVO MA 10 (“Carl Zeiss AG”, Oberkochen, BW, Germany) with energy-dispersive X-ray spectroscopy (“Bruker Nano GmbH”, Berlin, Germany). Hardness was measured by nanoindentation, using a Shimadzu DUH-211S nanotester (Kyoto, Japan) with a Berkovich tip indenter in Loading–Unloading mode (10 mN force, 1.4632 mN/s loading speed, 5 s hold time at 22 °C). Therefore, the basic requirement that the maximum indenter penetration depth must not exceed 10% of the total coating thickness was met and the potential influence of the elastic response of the substrate on the measured values was eliminated.

To assess carbon bonding in the coatings, Raman spectroscopy was performed using a LabRAM HR Visible spectrometer (Horiba Scientific, Kyoto, Japan) with a 633 nm He-Ne laser (0.57 mW, ×50 objective) for a 10 s measurement time with 10 repetitions.

## 3. Results

**Figure 2:**
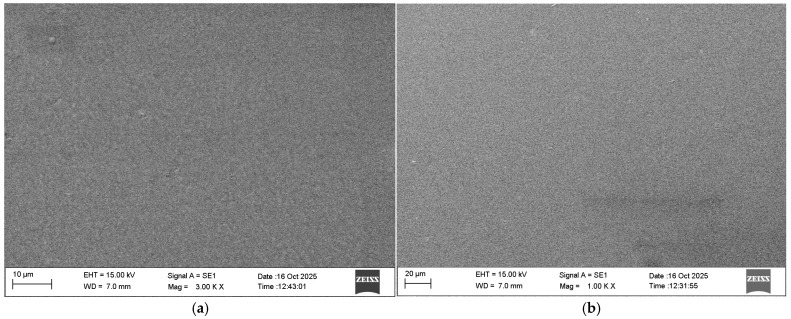
SEM images from the surface of nanostructured multilayer Cr-C coatings on AISI 316L (**a**) and AISI 321 (**b**) steel substrate.

The results from SEM analysis demonstrate that the obtained nanostructured multilayer Cr/(Cr/a-C)ml coatings on AISI 316L and AISI 321 steel substrates have a smooth and relatively homogeneous surface morphology. There are no large visible craters, micropores, cracks, or embedded large particles detached from the targets in the form of accumulated protruding clusters of Cr and graphite or other defects—Figure 2.

**Figure 3:**
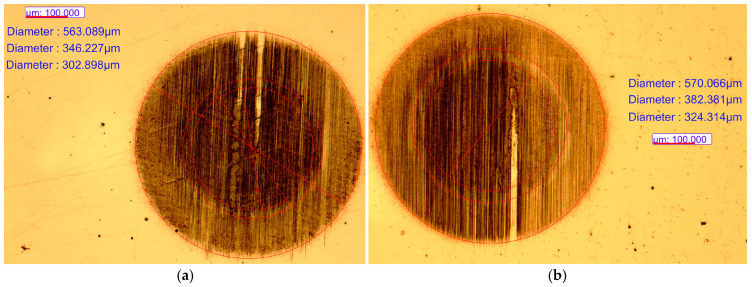
Measurements to determine the thicknesses of the individual layers after Calotest (**a**) on AISI 316L and (**b**) on AISI 321 steel substrate.

**Figure 4:**
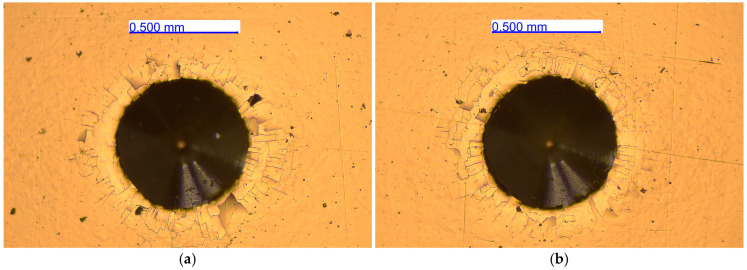
Evaluation of coating adhesion according to EN ISO 26443 for AISI 316L (**a**) and AISI 321 (**b**) steel substrate (×100).

The results represented in Table 3 are summarized from the protocols made according to the standards for both tests. To determine the thickness (EN ISO 26423:2016) of the coating, we performed five measurements per sample at Figure 3 and six measurements for adhesion class (EN ISO 26443:2024) at Figure 4. According to the manufacturer’s data of the BAQ GmbH (Germany), the measurement accuracy is dependent on the surface roughness of the coatings and its range is 1 ÷ 5% for the “KaloMAX II” calotester.

The nanostructured multilayer Cr/(Cr/a-C)ml coating was deposited for 180 min at 2 rpm rotation on the table with the substrates. Based on its thickness, given in Table 3, it was calculated with Equation (1) that the coating has a bi-layer period of Λ = 4.17 ± 0.39 nm on AISI 316L steel and Λ = 4.16 ± 0.18 nm on AISI 321.

(1) $$\Lambda =\frac{h}{t.r_{ts}}$$

where “h” is (Cr/a-C)ml coating thickness [nm], “t” is deposition time [min] and “r_ts_” is rotation on the table [min^−1^].

Most of the results from the adhesion test (EN ISO 26443) of the AISI 316 steel substrate coatings show cracking without adhesive delamination (class 1) but for two (from six) of them there is cracking with partial adhesive delamination (class 2).

EDX analysis gives the chemical composition of the obtained coatings on both stainless steel substrates, as well as the ratio of Cr/C atoms in the coating. The energy-dispersive X-ray spectroscopy was performed on at least 4 points for every sample (with a total of 24 points for both steel grades).

The results of the EDX analysis are represented as an average for every sample—for AISI 316L in Table 4 and for AISI 321 in Table 5.

**Figure 5:**
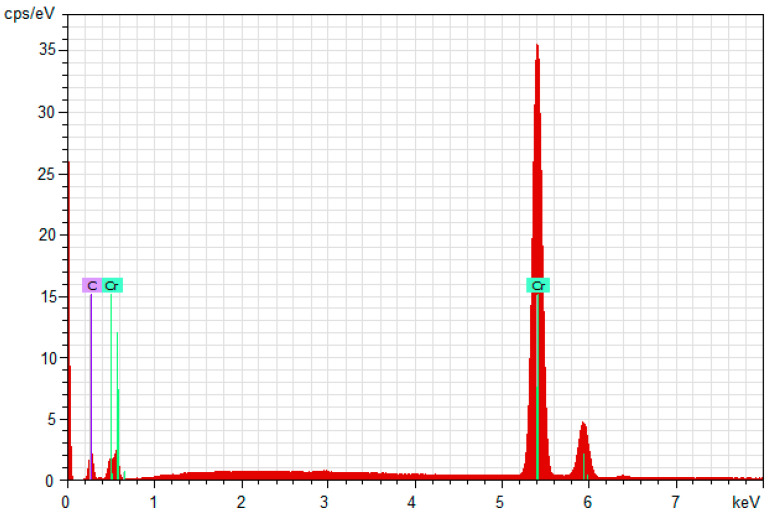
EDX spectroscopy from sample 1 with AISI 316L steel substrate.

**Figure 6:**
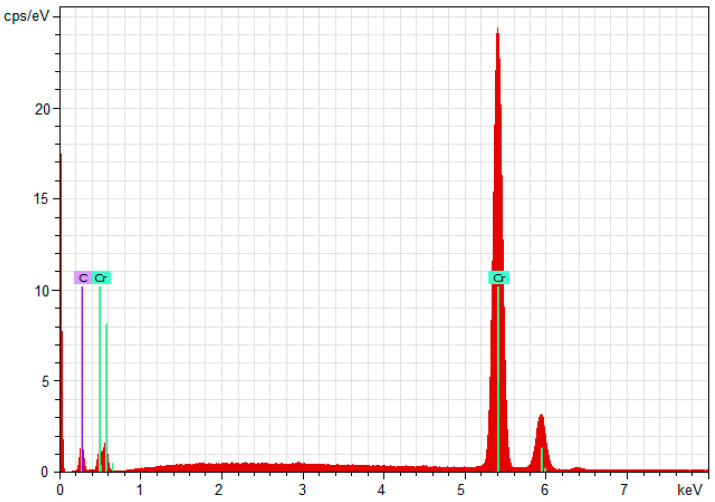
EDX spectroscopy from sample 3 with AISI 321 steel substrate.

The results obtained from the EDX analysis show the same chemical composition and C/Cr ratio in the coatings, with a high amount of chromium on both steel substrates. The resulting coating is free of impurities—no other chemical elements were registered in the coating except the deposited Cr and C shown at Figure 5 and Figure 6.

**Figure 7:**
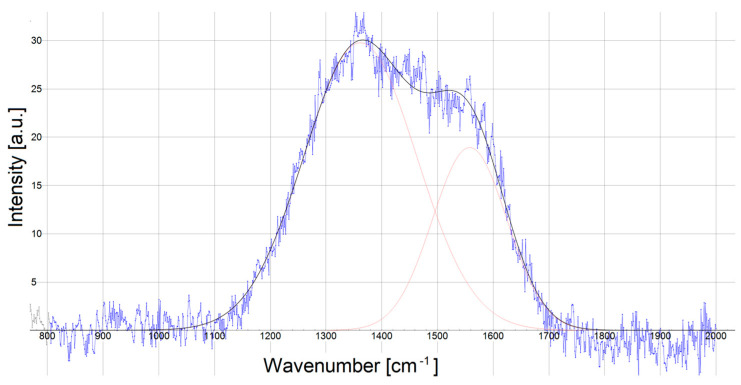
Raman spectrum of AISI 316L steel substrate.

**Figure 8:**
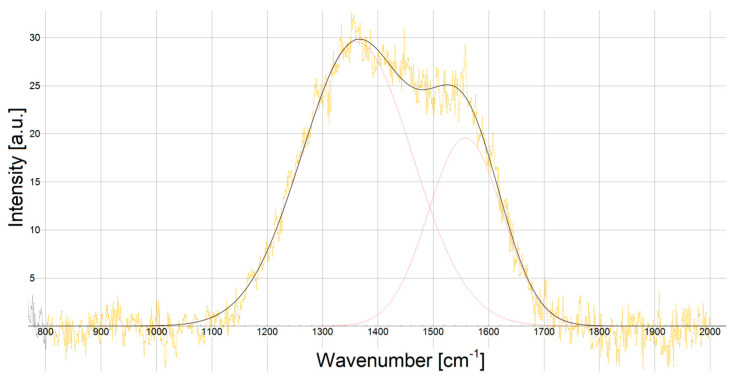
Raman spectrum of AISI 321 steel substrate.

The Raman-spectroscopic structural analyses of stainless steels 316L (Figure 7) and 321 (Figure 8) are presented below. For carbon-based coatings, the degree of carbon hybridization is a key determinant of their mechanical response. The Cr/(Cr/a-C)ml coatings exhibit broad D bands at 1362–1363 cm^−1^ and G bands at 1557–1558 cm^−1^, which is a signature of amorphous carbon containing mixed sp^2^ and sp^3^ configurations. The resulting sp^3^/sp^2^ ratio was extracted with fitted Gaussian profiles. The ratio, shown in Table 6, with values of 1.51 ÷ 1.57 was derived from the intensity ratio *I_D_/I_G_* obtained after deconvoluting the spectra. The results represent a clearly significant amount of diamond-like sp^3^ bonding, which underpins the high hardness expected from these films.

The mechanical properties of coatings are the most important characteristics for their applications. They were determined by nanoindentation using the Oliver and Pharr method [40,41]. The data are given in Table 7. The indicated values are averaged from 10 measurements. To comply with the requirement that the maximum depth of immersion of the indenter should not exceed 10% of the thickness of the measured coating, the indentation was performed with a load of Fmax = 10 mN. The table also shows the calculated elasticity index, resistance to plastic deformation index and resistance to crack formation index, which are adopted to predict these properties [42,43,44].

The measured values are averages with a tendency towards the upper range of the elastic–plastic indicators typical for these types of nanostructured coatings.

The conversions of the Vickers hardness HV*, indentation creep Cit, the elastic part of the indentation work η_it_, and the rate of elastic recovery of indentation R_er_ in Table 6 were calculated automatically by the Shimadzu DUH-211S software (ver.2.50) after the indentation procedure using the following formulas [45].

Conversion Vickers hardness HV*:

(2) $$HV*=0.0924H_{it}$$

where Hit is indentation hardness [MPa].

Indentation creep Cit %:

(3) $$C_{it}=\frac{\left(h_{2}-h_{1}\right)}{h_{1}}\cdot 100$$

where h_1_ is the indentation depth at the time of maximum force [µm] and h_2_ is the indentation depth at the holding time [µm].

The elastic part of the indentation work η_it_ % is calculated by using the following formula:

(4) $$\;\eta_{it}=\frac{W_{elast}}{W_{total}}\cdot 100$$

where

(5) $$W_{total}=W_{elast}+W_{plast}$$

In this case, W_total_ is the total mechanical work indicated during the indentation procedure [nJ], W_elast_ is the elastic part of the indentation work, [nJ], and W_plast_ is the plastic part of the indentation work [nJ].

The elastic recovery rate R_er_ is calculated using the following values and calculation formula:

(6) $$R_{er}=\frac{\left(h_{max}-h_{min}\right)}{h_{max}}\cdot 100$$

where: R_er_ is the elastic recovery rate [%], h_max_ is the displacement at the end of the load holding time [µm] and h_min_ is the displacement at reaching minimum test force [µm].

## 4. Discussion

In general, coatings obtained by magnetron sputtering on both grades of stainless steel have almost identical parameters and properties. SEM analysis shows that the coatings on both types of substrate are homogeneous. This is confirmed by the low deviation values of the characteristics measured by nanoindentation; therefore, their indicators are similar. There is no significant difference between their thickness and chemical composition, which is also reflected in their mechanical properties. EDX analysis shows that the chemical composition of the coatings on both grades of steel is the same and consists of only about 67.5 at% chromium and 32.5 at% carbon. Therefore, diffusion of Fe or Ni from the substrates, as well as O_2_, H_2_, or N_2_ from the gas environment, is not detected in the coatings.

However, a more detailed review of the results obtained from this study shows a few characteristics of each of the coatings. The coating on AISI316L steel has slightly lower homogeneity (Figure 2a), and a smaller total thickness and Cr sublayer thicknesses (Table 3). This may be due to a more roughly prepared surface of the substrates, which leads to the creation of larger and less evenly distributed island-like crystallization centers on the pure Cr sublayer, hence its slower growth and smaller thickness of 0.182 µm compared to the 0.251 µm coating on AISI 321 steel. Furthering growth of the Cr/(Cr/a-C)ml layers, the growth rates on the two types of steel substrates became equalized because the difference in the average thicknesses for the (Cr/a-C)ml bi-layer is only 4 nm, while the difference in the thicknesses of the pure Cr sublayer is 69 nm. This can be explained by the smoothing effect of the magnetron sputtering process. As a result, the total thickness of the coating on AISI 316L steel is smaller. This proves the importance of the smoothness of the substrate not only for homogeneity, but also for the growth rate of the layers and for the mechanical characteristics, obtained from nanoindentation tests.

The measured hardness values (by nanoindentation), H_it_ = 21–23 GPa and Young’s Modulus of E_it_ = 343–344 GPa for the Cr/(Cr/a-C)ml coatings, are comparable to or higher than those reported by other researchers. The hardness values according to other authors are as follows:

- H_it_ = 9.7–12.1 GPa [28];
- H_it_ = 5.8–7.8 GPa and Eit = 50–140 GPa [35];
- H_it_ = 12.5–22.7 GPa and Eit = 110–240 GPa [19];
- H_it_ = 9–12 GPa и Eit = 85–155 GPa [46];
- H_it_ = 10.5–13.5 GPa [47].

The high hardness and Young’s Modulus of the obtained Cr/(Cr/a-C)ml coatings can be explained by the high content of diamond-like carbon in them. This is proven by the high intensity ratio *I_D_/I_G_* =1.51 ÷ 1.57, where the D peak may also be associated with the sp^3^ carbon phase, which indicates the possible presence of DLC in the coating. At intensity ratio *I_D_/I_G_* =1.51 ÷ 1.57, it is logical to conclude that the values of H_it_ and E_it_ are even higher, but, the presence of Cr in the coating, along with the graphite phase, caps the hardness and Young’s Modulus to the measured values.

The coating on the AISI 321 steel substrate has a more homogeneous structure and this contributes to the higher values of hardness and modulus of elasticity. At the same time, quite logically, the high value of indentation creep (Cit) of the coating on AISI 316L steel is an indicator of a higher degree of relaxation of the internal stresses compared to the coating on AISI321 steel. This is especially important if these coatings are applied to precision parts or tools, e.g., for cameras and other finely detailed mechanisms.

The homogeneity of the substrate microstructure also affects the adhesion of the coatings. Coatings on AISI 316L steel substrates, which have more inhomogeneous structures, have weaker adhesion, as shown in Table 3. A way to improve its adhesion is to provide a smoother surface on the substrate before applying the coating, which would also lead to improvement in the mechanical characteristics of the deposited coating. The other way is to adjust the sublayer more precisely to reduce the layer and the layer–substrate internal stresses, in which case the sublayer should relax. The wide capabilities and process flexibility of the magnetron sputtering system used allow this problem to be studied for the specific Cr/(Cr/a-C)ml coatings and substrates. A way should be found to increase the hardness and elastic modulus of the nanostructured multilayer Cr/(Cr/a-C)ml coatings deposited on the two stainless steel grades studied, which would expand the possibilities for their applications. The reason for this is also the amorphous state of carbon with mixed sp^2^ and sp^3^ bonding that was registered in the coatings with a high intensity ratio *I_D_/I_G_* =1.51 ÷ 1.57. The presence of diamond-like sp^3^ phases in the coating, in combination with the chromium carbide in it, provides potential for searching for new process parameters to obtain coatings with even higher hardnesses and moduli of elasticity. According to other articles, diamond-like carbon (DLC) PVD coatings with a higher ratio of sp^3^ to sp^2^ carbon bonds typically exhibit greater nanohardness. Raman spectroscopy, specifically the analysis of the D and G bands and their ratio, is a key method used to characterize the *I_D_*/*I_G_* content and correlate it with mechanical properties like hardness [37,48,49,50].

The innovation in the current paper, compared with paper [37], is the substrate, which in our case is low-carbon austenite stainless steel and the number of Cr-C layers in the coating. The substrates used in paper [37] are quenched tool steel with heterogenous structure and higher hardness. These two factors—hardness and homogeneity of the microstructure of the substrate—influence the properties of the coating applied over it by magnetron sputtering, as homogeneity and types of (micro-)crystallites of the surface are the foundation (seeds) for growth of the coating’s phases and its uniformity or irregularity. On the other hand, stainless steels have high Cr content and it is assumed that this improves the adhesion with the Cr in the coating. A third consideration is that the substrates of AISI316L and AISI321 are not quenched (hardened), which is a predisposition for partial relaxation of some of the internal strain in the coatings as well as the softer substrate matter in contact loading over the coating (acting as a cushion). The most important difference is that the steels AISI316L and AISI321 are stainless and if the adhesion is improved then there are investigations to be made concerning the anti-corrosion behavior of these steels with and without the application of this particular coating.

## 5. Conclusions

Multilayer Cr/(Cr/a-C)ml coatings with a thickness between 1.68 µm and 1.75 µm were successfully obtained and deposited on AISI316L and AISI321 stainless steel substrates by unbalanced magnetron sputtering.

The resulting coatings contain 360 binary Cr/C layers with a bi-layer period of Λ = 4.16 ÷ 4.17 nm, which proves that they are nanostructured.

The obtained multilayer coatings Cr/(Cr/a-C)ml have a homogeneous surface, free of defects and with a chemical composition consisting only of C and Cr, without impurities from other chemical elements, which has been proven by EDX analysis. The coatings have a hardness and modulus of elasticity from nanoindentation of 21 ÷ 23 GPa and 343 ÷ 344 GPa, respectively, which make them suitable for a wide range of applications in various fields of industry, medicine, energy, etc.

The results obtained from this work validate the magnetron sputtering and process parameters used for deposition of nanostructured multilayer Cr/(Cr/a-C)ml coatings on AISI316L and AISI321 stainless steels as suitable.

## Figures and Tables

**Table 1:** Chemical composition of steels, wt%.

Steel	C	Si	Mn	Ni	P	S	Cr	Mo	N	Ti
AISI 316L	≤0.03	≤1	≤2	10–13	≤0.045	≤0.015	16.5–18.5	2–2.5	≤0.11	-
AISI 321	≤0.08	≤1	≤2	9–12	≤0.045	≤0.015	17–19	-	≤0.10	≤0.7

**Table 2:** Test-specific parameters for the determination of coating thickness according to EN ISO 26423:2016.

№	Test Parameters	Values
1	ball diameter	30 mm
2	contact load	0.54 N
3	rotational speed of ball	200 rpm
4	composition of the abrasive slurry	1 µm diamond paste in ethanol
5	slurry feed rate	1 drop/20 s
6	duration of the test	20 s

**Table 3:** Results from the thickness and adhesion tests of Cr/(Cr/a-C)ml PVD coating.

Substrate Material	Cr Sublayer Thickness	(Cr/a-C)ml Coating Thickness	PVD Coating Total Thickness	Adhesion Class EN ISO 26443
AISI 316L	0.182 ± 0.053 µm	1.502 ± 0.141 µm	1.684 ± 0.193 µm	Class 1(2)
AISI 321	0.251 ± 0.091 µm	1.498 ± 0.065 µm	1.749 ± 0.123 µm	Class 1

**Table 4:** Element content in the (Cr/a-C)ml coating on the AISI 316L steel substrate.

Element	Average at% Sample 1	Average at% Sample 2	Average at% Sample 3	Average at% Coating
Cr	67.13	67.09	67.32	67.18
C	32.87	32.91	32.68	32.82

**Table 5:** Element content in the (Cr/a-C)ml coating on the AISI 321 steel substrate.

Element	Average at% Sample 1	Average at% Sample 2	Average at% Sample 3	Average at% Coating
Cr	67.45	67.98	68.15	67.86
C	32.55	32.02	31.85	32.14

**Table 6:** Raman spectrum results of stainless steel substrates.

AISI 316L		AISI 321	
**Center**	**Intensity**	**Center**	**Intensity**
1361.6	29.777	1362.3	29.544
1557.9	18.928	1557.9	19.571
*I_D_/I_G_* (Int) = 1.57		*I_D_/I_G_* (Int) = 1.51	

**Table 7:** The mechanical properties of the coatings determined by nanoindentation.

Properties		Substrate	
		AISI 316L	AISI 321
Maximum indentation depth at Fmax	h_max_	0.11493 ± 0.0020 µm	0.1461 ± 0.0030 µm
Indentation hardness	H_it_	21.683 ± 1.029 GPa	23.084 ± 1.176 GPa
Indentation modulus	E_it_	343.5 ± 12.6 GPa	344.3 ± 10.2 GPa
Indentation creep	C_it_	2.858 ± 0.735%	2.248 ± 0.811%
Elastic part of indentation work	η_it_	52.784 ± 3.09%	53.394 ± 3.43%
Conversion Vickers hardness from Hit	HV*	2003.55 ± 95.15	2132.96 ± 108.73
Rate of elastic recovery of indentation	R_er_	48.326 ± 3.031%	47.438 ± 1.833%
Elasticity index	H_it_/E_it_	0.063	0.067
Resistance to plastic deformation	H_it_^3^/E_it_^2^	0.086	0.103
Resistance to crack formation index	1/H_it_.E_it_^2^	3.91 × 10^−7^	3.65 × 10^−7^

## Data Availability

The original contributions presented in this study are included in the article. Further inquiries can be directed to the corresponding authors.

## Abbreviations

PVD	Physical vapor deposition
Cr/(Cr/a-C)ml	Chromium/amorphous carbon multilayer coating
DLC	Diamond-like carbon coating
EDX	Energy-dispersive X-ray analysis
SEM	Scanning electron microscope
MFP	Medium frequency power
F_max_	Maximum test force of indentation
h_max_	Maximum indentation depth at Fmax
H_it_	Indentation hardness
E_it_	Indentation modulus
C_it_	Indentation creep
η_it_	Designation of elastic part of indentation work
HV*	Conversion Vickers hardness from Hit
R_er_	Rate of elastic recovery of indentation
